# Simulated Microgravity Promotes Horizontal Gene Transfer of Antimicrobial Resistance Genes between Bacterial Genera in the Absence of Antibiotic Selective Pressure

**DOI:** 10.3390/life11090960

**Published:** 2021-09-13

**Authors:** Camilla Urbaniak, Tristan Grams, Christopher E. Mason, Kasthuri Venkateswaran

**Affiliations:** 1 NASA Jet Propulsion Laboratory, California Institute of Technology, Pasadena, CA 91109, USA; camilla.urbaniak@jpl.nasa.gov (C.U.); tgrams@ufl.edu (T.G.); 2 ZIN Technologies Inc., Middleburg Heights, OH 44130, USA; 3 Department of Physiology and Biophysics, Weill Cornell Medicine, New York, NY 10065, USA; chm2042@med.cornell.edu; 4 The World Quant Initiative for Quantitative Prediction, Weill Cornell Medicine, New York, NY 10065, USA

**Keywords:** horizontal gene transfer, simulated microgravity, high aspect ratio vessel (HARV), antimicrobial resistance, space environment, *Staphylococcus aureus*, *Acinetobacter pittii*

## Abstract

Bacteria are able to adapt and survive in harsh and changing environments through many mechanisms, with one of them being horizontal gene transfer (HGT). This process is one of the leading culprits in the spread of antimicrobial resistance (AMR) within bacterial communities and could pose a significant health threat to astronauts if they fell ill, especially on long-duration space missions. In order to better understand the degree of HGT activity that could occur in space, biosafety level-2, donor and recipient bacteria were co-cultured under simulated microgravity (SMG) on Earth with concomitant 1G controls. Two AMR genes, *bla*_OXA-500_ and *ISAba1,* from the donor *Acinetobacter pittii,* were tracked in four recipient strains of *Staphylococcus aureus* (which did not harbor those genes) using polymerase chain reaction. All four *S. aureus* strains that were co-cultured with *A. pittii* under SMG had a significantly higher number of isolates that were now *bla*_OXA-500_- and *ISAba1*-positive compared to growth at 1G. The acquisition of these genes by the recipient induced a phenotypic change, as these isolates were now resistant to oxacillin, which they were previously susceptible to. This is a novel study, presenting, for the first time, increased HGT activity under SMG and the potential impact of the space environment in promoting increased gene dissemination within bacterial communities.

## 1. Introduction

Bacteria are able to adapt to a wide variety of conditions, as evidenced by their survival and growth in extreme environments, such as the arid Atacama desert [1], hypersaline salt mines and lakes [2,3], acidic rocks in the Yellowstone geothermal environment [4], and the multi-stressor environment of Antarctica [5]. Bacterial communities have also been found beyond the Earth’s surface on the International Space Station (ISS), a low-gravity, high-radiation, hermetically sealed built environment [6]. The microbial communities characterized here consist of environmental organisms, human-associated commensal bacteria and biosafety level 2 (BSL-2) microbes, resistant to various antibiotics, and carrying numerous anti-microbial resistance genes [7,8,9]. This resistance could potentially pose a serious threat to astronauts if they fell ill, especially on long-duration space missions, as medical attention would not be readily available.

Horizontal gene transfer (HGT) is the movement of genetic material (i.e., DNA) between microorganisms through either transformation, conjugation or transduction, and is considered to be central to microbial evolution [10,11]. In dynamic mixed communities, either human or environmental, HGT between species may confer an overall fitness advantage to the community, as the acquisition of new traits in individual cells within that community allows them to better adapt to changing conditions [12,13,14,15]. In other cases, HGT may have an unfavorable effect if it leads to an excessive accumulation of genes, or a neutral one [16], if there is no impact on the fitness of the organism [16].

In a recent study comparing bacteria from two extreme built environments, one on Earth (Concordia Research Station in Antarctica) and one in space (International Space Station), a comparison between isolates of the same species showed that those from the International Space Station (ISS) had more mobile genetic elements within their genomes than those from Concordia [17]. In a follow-up analysis conducted by our group using the ISS resistome and microbiome data presented by Urbaniak et al. 2018 [7], we performed a series of correlation analyses to determine which antimicrobial resistance (AMR) genes were associated with which species. Interestingly, no statistically significant correlations were found. These data, coupled by the Concordia/ISS study, led us to hypothesize that the stressors of the ISS, specifically microgravity, promote enhanced HGT between organisms, compared to what would be observed on Earth.

To address this hypothesis, co-cultures between donor and recipient strains were grown under simulated microgravity (SMG) (which we used as a proxy for µgravity conditions on the ISS) and normal gravity (1G), using the high-aspect-ratio vessel (HARV), with gene transfer tracked using polymerase chain reaction (PCR). The donor and recipient strains were *Acinetobacter pittii* (strain IIF1SW-P1) and *Staphylococcus aureus* (strains IF4SW, IIF6SW, IF7SW, IIF8SW), respectively. All strains were previously isolated from various locations on the ISS as part of the Microbial-1 Tracking study [8]. *A. pittii* IIF1SW-P1 was chosen as the donor, since it was previously shown by our group to be resistant to oxacillin and contained the chromosomally located *bla*_OXA-500_ gene within its genome [7], a beta-lactamase which alone can confer resistance to oxacillin [18]. The four *S. aureus* strains, IF4SW, IIF6SW, IF7SW, IIF8SW, were chosen as recipients because they were susceptible to oxacillin and harbored neither the *bla*_OXA-500_ gene nor any other beta-lactamase genes [7]. The donor, *A. pittii,* harbored *ISAba1*, a transposable element that can jump between segments of the genome and, in some cases, can act as a promoter region for *bla*_OXA_ genes, allowing for increased resistance to oxacillin and other beta-lactam antibiotics [19,20]. *ISAba1* was not found in either of the recipient *S. aureus* strains. HGT ability was, therefore, tracked between *A. pittii* and *S. aureus* using the AMR genes, *bla*_OXA-500_ and *ISAba1*.

## 2. Methods

### 2.1. Co-Culture Experiments

All strains examined in this study were cultivated from the ISS environment collected during Flight 2 (May 2015) as per the published protocol [8]. *A. pittii* strain, IIF1SW-P1, was isolated at Location #1, the Port panel of the Cupola. The Cupola is a small module devoted to the observation of operations outside the ISS, such as robotic activities, spacecraft approaches, and extravehicular activities. *S. aureus* strain IF4SW was isolated from Location #4, the surface of the dining table. Even though the main function of the table was for dining, crew members also used the table for experimental work. *S. aureus* strain IIF6SW was cultured from the sample collected at Location #6, a stowage rack where experimental materials were stored for long duration. *S. aureus* strain IF7SW was isolated from Location #7, an overhead three-panel surface of the Materials Science Research Rack 1, which is used for basic material research in the microgravity environment of the ISS. *S. aureus* strain IIF8SW was retrieved from the samples at Location #8, an exterior aft wall of crew quarters. The crew quarters are a permanent personal space for crewmembers to sleep and perform personal recreation and communication, as well as provide on-orbit stowage of personal belongings.

Donor (*A. pittii* strain IIF1SW-P1) and recipient (*S. aureus* strains IF4SW-P1, IIF6SW-P1, IF7SW-P3, IIF8SW-P1) bacteria were streaked from −80 °C freezer stocks onto tryptic soy agar plates (Hardy Diagnostics, Santa Maria, CA, USA) and incubated overnight (~16 h) at 37 °C. A single colony from each plate was added to 10 mL of tryptic soy broth (TSB) in a 50 mL Falcon tube for *S. aureus*, and 8 mL of TSB in a snap cap tube for *A. pittii* and grown overnight (~20 h) in a shaking incubator at 85 rpm. A low speed was chosen, so as to not dislodge pili or flagella that could be used for HGT, but high enough that the bacteria did not settle out of culture over time. The overnight culture was centrifuged for 10 min at 3000× *g*, the supernatant decanted, and the pellet washed in 1× sterile phosphate-buffered saline (PBS). Pellets were then resuspended in 10 mL of 1× PBS. For *S. aureus*, 1 mL was then transferred to 9 mL of PBS and, for *A. pittii*, the tube was topped with 3 mL of PBS. The biomass of the cultures was measured with DENSICHEK^®^ (bioMérieux, Marcy-l’Étoile, France) and adjusted with PBS to reach a concentration of 10^8^ cfu/mL, which was then serially diluted to 10^6^ cfu/mL. 100 µL of one *S. aureus* recipient strain was added to 10 mL of TSB and 100 µL of the donor *A. pittii* was added to the same 10 mL tube to set up the co-cultures. After gentle mixing, the complete 10 mL volume containing the donor and recipient was carefully added to HARVs to avoid any bubbles. HARVs were grown under SMG (vertical position) and 1G (horizontal position) at 33 rpm for 20 h at 37 °C.

### 2.2. Isolation of Colonies after HARV Growth

After incubation, the liquid from each HARV vessel was collected and 100 µL, in duplicate, was plated on MSA (selective for *S. aureus*) (VWR, Radnor, PA, USA), MSA+ 4 µg/mL oxacillin sodium salt monohydrate (VWR, USA) (selective for *S. aureus* that had become resistant to oxacillin) and Leeds agar (selective for *A. pittii*) (VWR, USA). MSA and Leeds plates were incubated overnight at 37 °C and MSA+ oxacillin plates were incubated for 72 h at 37 °C. Colonies on MSA, MSA+ oxacillin, and Leeds plates were counted, and cfu/mL was calculated. Colonies that grew on MSA+ oxacillin plates were picked and placed in 250 µL 1× PBS and stored at 4 °C until DNA extraction. DNA was extracted using the ZymoBIOMICS DNA Microprep Kit (ZymoResearch, Irvine, CA, USA) following the manufacturer’s instructions.

### 2.3. Designing Primers

Primers were designed using the *A. pittii* IIF1SW-P1 draft genome that we had previously published, in which *bla*_OXA-500_ gene resides on scaffold 5 and *ISAba1* gene on both scaffolds 44 and 70 [21]. Primers were designed using IDT DNA PrimerQuest for genes of interest. IDT DNA OligoAnalyzer was used to ensure no hairpins and correct PCR melting temperature. NCBI BLAST was used to ensure no off-target binding sites for strains used in this study. Amplification product of the primers for *bla*_OXA-500_ and the location within the genome is shown in Figure S3A and for *ISAba1*, in Figure S3B in Supplementary Materials. The product size of *bla*_OXA-500_ is 591 bp and for *ISAba1*, is 390 bp.

### 2.4. PCR

Each 25-μL reaction consisted of 12.5 μL of GoTaq^®^ Green Master Mix (Promega, Madison, WI, USA), 1 μL each of forward and reverse oligonucleotide primers (10 μM each), 5.5 µL of nuclease free water (Promega, USA) and 5 μL of template DNA. The following primer pairs were used:

- *bla*_OXA-500__F (5′-CCGAGTTGTTCCAATCCCTTAT-3′) and
- *bla*_OXA-500__R (5′-ATATGTTCCCGCCTCTACCT-3′) to amplify *bla*_OXA-500_
- and *ISAba1*_F (5′-ATGCAGCGCTTCTTTGCAGG-3′) and
- *ISAba1*_R (5′-AATGATTGGTGACAATGAAG-3′) to amplify *ISAba1*.

The reaction conditions were as follows: 95 °C for 5 min, 35 cycles of 95 °C for 30 s, 55 °C for 30 s, 72 °C for 1 min and then a final extension of 72 °C for 5 min. Amplified PCR products were run on the E-Gel™ Power Snap Electrophoresis System (Thermo Fischer, Waltham, MA, USA, cat # G8300) using their pre-cast 1.2% SYBR Safe, E-gels (cat # G521801). Gels were run with a 1 Kb plus DNA ladder (cat # 10488090). Gel images were captured with the built-in camera. The positive control consisted of DNA isolated from the parental *A. pittii* strain and the negative controls were DNA isolated from the parental *S. aureus* strains. A no-template control (NTC) consisting of molecular-grade water instead of DNA was used.

For PCR reactions that were negative, a follow-up PCR was performed using the 16S rRNA gene to confirm that the DNA extraction procedure produced sufficient DNA. The following primers were used for 16S rRNA gene amplification: the forward primer, 27F (5′-AGA GTT TGA TCC TGG CTC AG-3′) and the reverse primer, 1492R (5′-GGT TAC CTT GTT ACG ACT T-3′). The PCR conditions were as follows: denaturation at 95 °C for 5 min, followed by 35 cycles consisting of denaturation at 95 °C for 50 s, annealing at 55 °C for 50 s, and extension at 72 °C for 1 min 30 s and finalized by extension at 72 °C for 10 min. PCR with the 16S rRNA gene showed that all our samples did indeed have the DNA product and that extraction was successful.

### 2.5. Calculating the Efficiency of Gene Transfer

The number of colonies that had grown on MSA+ oxacillin and were positive for at least one gene (*bla*_OXA-500_ or *ISAba1*) was divided by 0.1 mL (the volume of the HARV culture used for plating). This number represents the theoretical concentration (cfu/mL) of cells in the culture that have acquired one of the genes. This number was then divided by the total concentration of *S. aureus* cells (cfu/mL) in the culture, which was calculated from the number of colonies that grew on MSA.

### 2.6. Chromosome vs. Plasmid Location of bla_OXA-500_

Others have documented in the literature that *bla*_OXA-500_ is located on the chromosome and is not a plasmid. To confirm that the same was true for our donor strain, *A. pittii* IIF1SW-P1, the following analyses were performed. plasmidSPAdes was used to assemble plasmid sequences from the whole genome-sequencing data of our strain. A simple blastn comparison between the *bla*_OXA-500_ sequence and the plasmid sequences showed the absence of the gene from the plasmid-assembled contigs. Furthermore, oriTfinder was used to determine whether scaffold_5 (length = 172 kb), on which *bla*_OXA-500_ was found, as well as the assembled sequences not characterized as plasmid sequences (from the plasmidSPAdes output above), contained evidence of plasmid markers, such as an origin of transfer site (*oriT*) and relaxases. None of these plasmid markers were detected on scaffold_5 and the “chromosomal” sequences.

## 3. Results

In order to determine whether HGT occurred between donor and recipient, suitable PCR primers were designed to amplify regions of the *bla*_OXA-500_ and *ISAba1* genes from *A. pittii* IIF1SW-P1. The sequences of the two genes, used to design the primers, were extracted from the draft whole-genome sequence of *A. pittii* IIF1SW-P1 [21]. PCR analysis performed on DNA isolated from *A. pittii* IIF1SW-P1 using the designed *bla*_OXA-500_ and *ISAba1* primers showed strong bands on an agarose gel, while PCR analysis performed on DNA isolated from the four *S. aureus* strains showed no amplicons.

**Figure 1:**
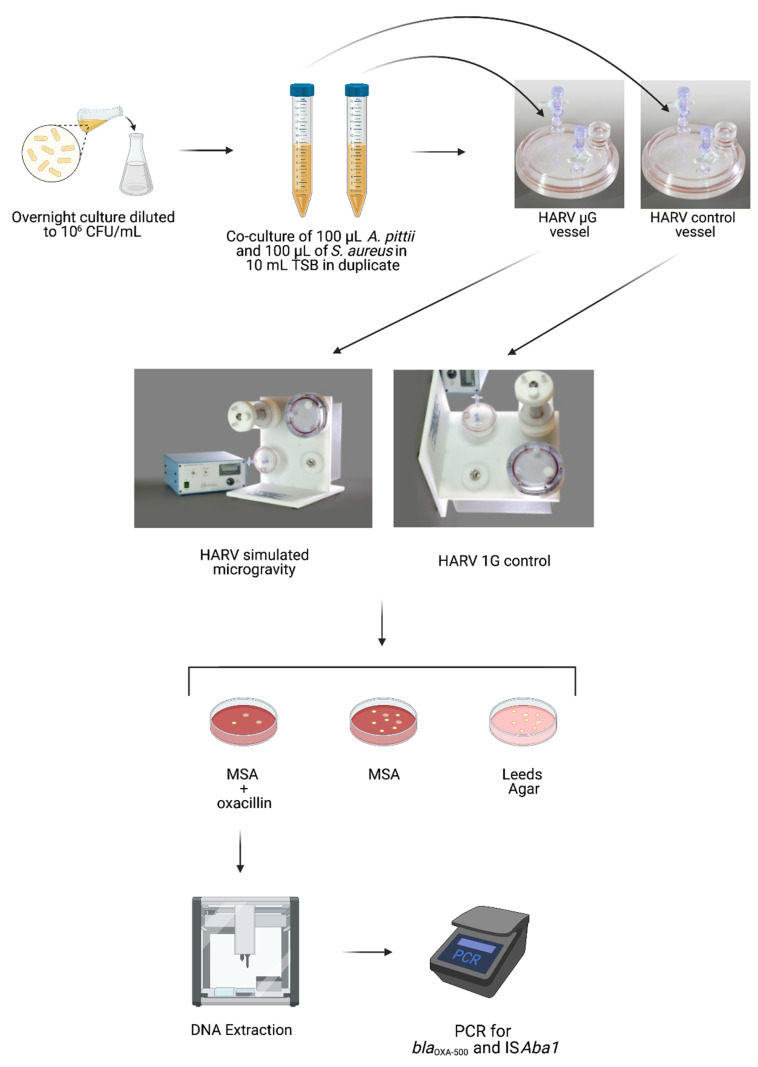
Schematic of experimental protocol.

To compare HGT potential between SMG and 1G, co-cultures of *A. pittii* (donor strain) and *S. aureus* (recipient strain) were grown in the HARV under SMG (vertical rotation) or 1G (horizontal rotation) for 20 h, after which the culture was plated on mannitol salt agar (MSA) to select for *S. aureus*. The MSA was also supplemented with oxacillin to select only those *S. aureus* colonies that had acquired resistance to the antibiotic. To verify that this newly acquired resistance to oxacillin was not due to spontaneous mutations but rather through the HGT of *bla*_OXA-500_ and *ISAba1* from *A. pittii*, the *S. aureus* colonies that grew on MSA supplemented with oxacillin were picked for PCR analysis. A schematic of the experimental set-up is shown in Figure 1.

**Figure 2:**
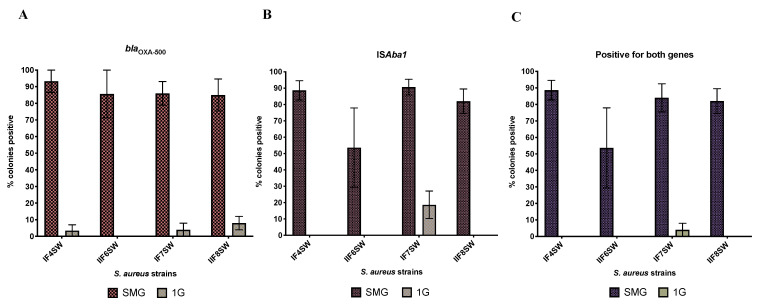
Comparison of HGT activity between SMG and 1G. Four strains of *S. aureus* (IF4SW, IIF6SW, IF7SW, IIF8SW) were used as the recipient and were co-cultured (separately) with the donor strain *A. pittii* for 20 h at 37 °C under SMG or 1G conditions. Cultures were plated on MSA containing 4 µg/mL of oxacillin. Colonies that grew were picked, subjected to DNA extraction and then analyzed by PCR with primers specific to *bla*_OXA-500_ and *ISAba1* from the donor strain. The graphs show the percent of *S. aureus* colonies, from each strain, that grew on MSA+ oxacillin that had acquired (**A**) *bla*_OXA-500_, (**B**) *ISAba1* or (**C**) both genes from the donor *A. pittii*.

Figure 2 shows the proportion of colonies that grew on MSA supplemented with oxacillin that were positive for *bla*_OXA-500_ (Figure 2A), *ISAba1* (Figure 2B) and both (Figure 2C). Representative gel images are shown in Figure S1. On average, 77% of the *S. aureus* colonies that were grown with *A. pittii* under SMG and had acquired resistance to oxacillin now harbored both *bla*_OXA-500_ and *ISAba1* (89% of the colonies had acquired at least one). This is in contrast with 1G, where only 1% of *S. aureus* colonies that had acquired oxacillin resistance were positive for both *bla*_OXA-500_ and *ISAba1* (8% of the colonies had acquired at least one).

The differences in proportions observed between SMG and 1G were not due to differences in growth at these different conditions, as each strain showed the same biomass after 20 h, whether grown under SMG or 1G (Figure S2).

The efficiency of transfer under SMG for all four strains was, on average, 3.06 × 10^−7^ while, for 1G, it was 3.21 × 10^−9^ (Table 1). While all strains had similar efficiencies, IIF8SW had the highest and IIF6SW the lowest.

To determine how stable these donor genes were in the recipient, two resistant *S. aureus* 1F4SW colonies that had acquired both genes were sub-cultured on MSA + oxacillin plates, a total of four times. Even after the 4th sub-culture, the colonies were still resistant to oxacillin, and PCR confirmed that *bla*_OXA-500_ and *ISAba1* were still present within the genome.

## 4. Discussion

We have shown that HGT is increased under SMG compared to 1G. The transfer of AMR genes, which were tracked by PCR, occurred in the absence of selective pressure by antibiotics. This transfer led to functional changes, as the acquisition of the two AMR genes, *bla*_OXA-500_ and *ISAba1*, that were tracked in the four strains of *S. aureus*, made them resistant to oxacillin, to which they were previously susceptible. These results have significant implications for long-duration space missions because pathogens that are susceptible to antibiotics could become resistant and thus harder to treat. In addition, even if astronauts are screened for problematic microbes prior to flight, issues could still arise, as commensals are known to act as reservoirs for AMR genes that can be disseminated to (opportunistic) pathogens [22,23,24]. For example, a study conducted in a hospital in the Netherlands showed that *mecA* gene transfer occurred in a patient between *mecA*^+^ *S. epidermidis* and a *mecA*^−^ strain of *S. aureus* [25].

It was beyond the scope of the study to determine the cause of the increased HGT that was observed, but one mechanism could be increased competence, allowing for transformation to occur, one of three modes of gene transfer. Competence allows bacterial cells to uptake DNA from its environment and increase the response to different stressors [26,27,28,29]. In this case, naked DNA released from dead *A. pittii* cells could have been readily taken up by *S. aureus* when exposed to SMG due to an increase in its competence. While *S. aureus* is not classically considered to be naturally competent, as *Streptococcus pneumoniae* and *Bacillus subtilis* are, it does harbor genes that are involved in competence and thought to promote natural competence under the right conditions [30,31]. The conditions that *S. aureus* needs are not yet known, but, similar to other bacteria, it could be an adverse environment. Indeed, when *S. aureus* cells were subjected to heat-shock, cold-shock and the stringent response, there was increased expression of competence gene orthologs compared to the non-stressed conditions [32]. In *S. pneumoniae* and *B. subtilis*, competence confers a survival advantage when cells are subjected to stress [33,34], so there is a possibility that growth under SMG led to an adverse environment for *S. aureus*, causing cells to become more competent to increase survival. Conjugation is a less likely scenario since, consistent with what others have documented in the literature, *bla*_OXA-500_ is located on the chromosome and not a plasmid. A second mechanism could be increased transduction caused by growth under SMG. Transduction is the transfer of genetic material via bacterial phages. It is well known that prophage activity is increased during stress, leading to increased phage production, helping the host survive these adverse conditions [35,36].

## 5. Conclusions

This paper describes, for the first time, the effects of SMG on HGT and has shown that it is increased in SMG compared to 1G. If the same trend is observed on the ISS, this will have a significant impact on how we view bacterial interactions in space and its effects on astronaut health. The next steps would be to elucidate the mechanisms behind the HGT, to develop appropriate countermeasures for long-duration space missions.

## Figures and Tables

**Table 1:** Efficiency of HGT. Average values of the three biological replicates, with the range of the values shown in parentheses. Efficiency was based on colonies that at acquired at least one gene (either *bla*_OXA-500_ or *ISAba1*).

*S. aureus* Recipient Strain	SMG (Range)	1G (Range)
IF4SW	4.21 × 10^−7^ (1.1 × 10^−6^ to 5.2 × 10^−8^)	2.05 × 10^−9^ (0.0 to 4.1 × 10^−9^)
IIF6SW	4.54 × 10^−8^ (7.5 × 10^−8^ to 6.3 × 10^−9^)	0.0 (no transfer)
IF7SW	3.20 × 10^−7^ (7.2 × 10^−7^ to 9.9 × 10^−8^)	4.33 × 10^−9^ (7.5 × 10^−9^ to 2.2 × 10^−9^)
IIF8SW	4.38 × 10^−7^ (6.8 × 10^−7^ to 3.1 × 10^−9^)	6.07 × 10^−9^ (0.0 to 1.2 × 10^−9^)

## Data Availability

The draft genomes of the parental strains used for the co-cultures can be found in NCBI under accession number MIZX00000000, MIZH00000000, MIZN00000000, MIZM00000000, MIZP00000000 and are presented in the manuscript by Checinska Sielaff A et al. [21].

## Supplementary Materials

The following are available online at https://www.mdpi.com/article/10.3390/life11090960/s1, Figure S1: Representative gel images of PCR analysis. Figure S2: Colony forming units of strains grown in SMG and 1G. Figure S3: Schematic of designed primers and their location within the donor genome.
