# Supplementary material for: Simulated Microgravity Promotes Horizontal Gene Transfer of Antimicrobial Resistance Genes between Bacterial Genera in the Absence of Antibiotic Selective Pressure
Source: Life (Basel). 2021 Sep 13;11(9):960. doi: 10.3390/life11090960 (PMC8468678; doi:10.3390/life11090960)

Supplementary figure captions

Figure S1. Representative gel images of PCR analysis when co-cultures were grown in simulated microgravity (SMG) or normal gravity (1G). Strong bands were visualized for both (A) $bla_{\mathrm{OXA\text{-}500}}$ and (B) IS*Aba1* amplification in *S. aureus* isolates when co-cultured with the donor under SMG. This was in contrast to the absence of bands when grown under 1G. "Ap" indicates the positive control which is DNA extracted from *A. pittii* parental strain. The no template PCR control (NTC) and DNA extraction negative control had no bands on the gels.

Figure S2. Colony forming units of strains grown in SMG or 1G. The graph shows the counts of *S. aureus* grown on MSA after growth in SMG or 1G, either on its own or in a co-culture with *A. pittii*. There were no differences in growth regardless of single or co-culture growth or under the different growth conditions. The same analyses were performed for *A. pittii* but the data is not shown.

Figure S3. Schematic of where the designed primers (A) $bla_{\mathrm{OXA\text{-}500}}$ and (B) IS*Aba1* are located in the *A. pittii* genome and the sequence of the PCR products.

Fig. S1

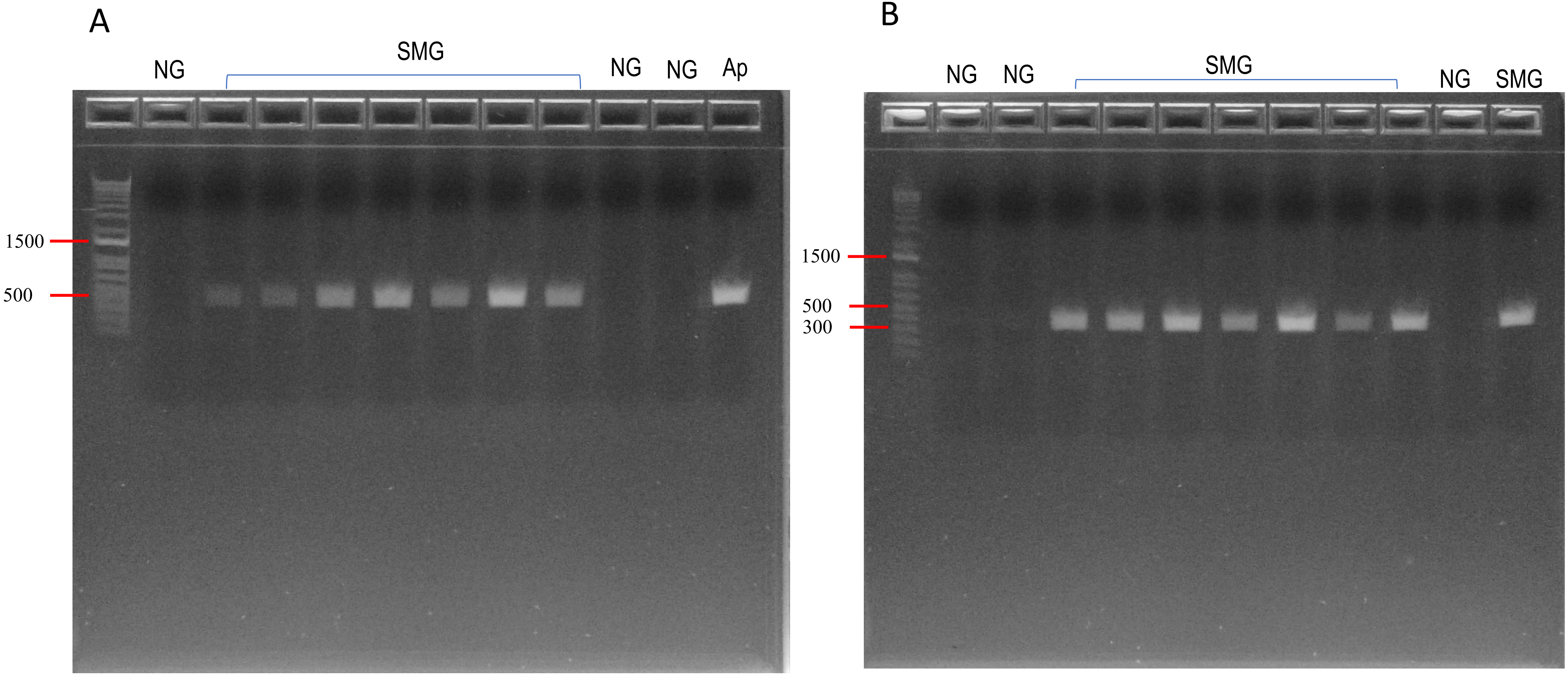

blaOXA500

Isaba1

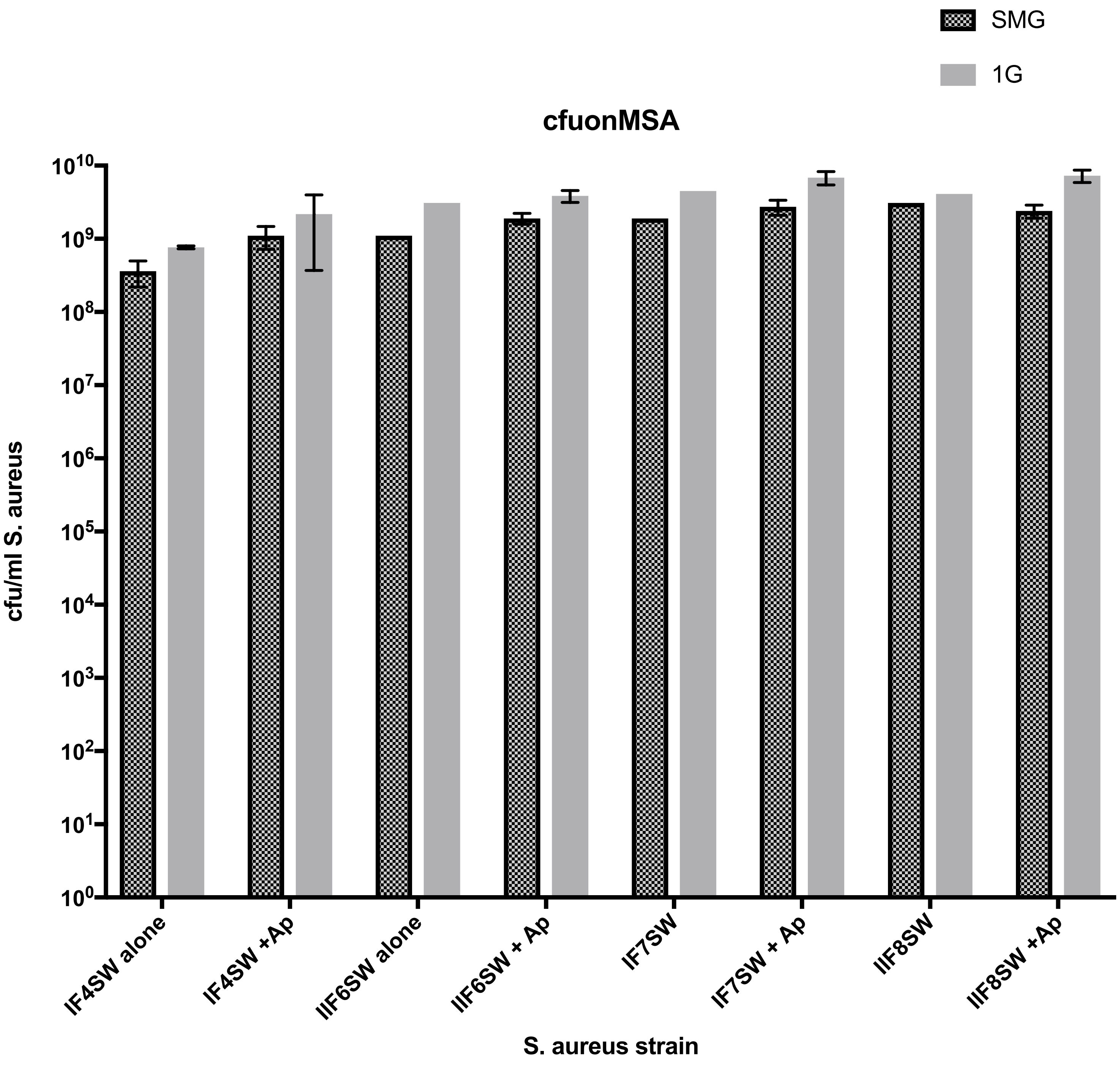
SMG
1G
cfuonMSA
cfu/ml S. aureus
10^10
10^9
10^8
10^7
10^6
10^5
10^4
10^3
10^2
10^1
10^0
IF4SW alone
IF4SW +Ap
IIF6SW alone
IIF6SW + Ap
IF7SW
IF7SW + Ap
IIF8SW
IIF8SW +Ap
S. aureus strain

Fig. S3A

Genomic location on Acinetobacter pittii strain IIF1SW-P1

GenBank: MIZX01000006.1

Forward: 6387-6408

Reverse: 6958-6977

PCR product expected size: 591 bp

Sequence:

CCGAGTTGTTCCAATCCCTTATAAGCAATTTCTTTTCGAATAGAGCTAGGTATGCCTTTTTTCATTTCTA
AATTAAGTGAGAAAGCTATAATTTCTCCTTGTGGTTGAACGACCCAGCCTGTTAACCAGCCCACTTGAGG
CTCCACATCCCATCCCCAACCACTTTTGGCATAAATTTTCCGTCCATTTTTTTCTTCTATGAACAGCATA
GATTGAACTTGTTCTTGCATATTTTTGCTAAAAGGAAGAGTTTTATGGGCTAGTTCATAAGCAAACTGGG
CTTCTTGTTGAGGTGTAATTTTAAGTGGACCAACAAGCCAAAAATCATCTACTTTTGAACCAATATCAGC
ATTACCGAAACCAATGCGTTTTACCTCTTTAGACATAAGATCAAGGCCAATTCGTCGAGCTAGTTCCTGA
TAAACTGGAATAGCAGAAGCTTTCATCGCATCGCCTAATGTCATATCTTTTTCCCAATCGGGAAATAAAC
GCTTTTGCCCATCCCATTTAAACACTTCAGTTGGTGTTGCTTTATGATGCTCAAGTCCAATCAAAGCATT
TAACATTTTAAAGGTAGAGGCGGGAACATAT

Primer Map:

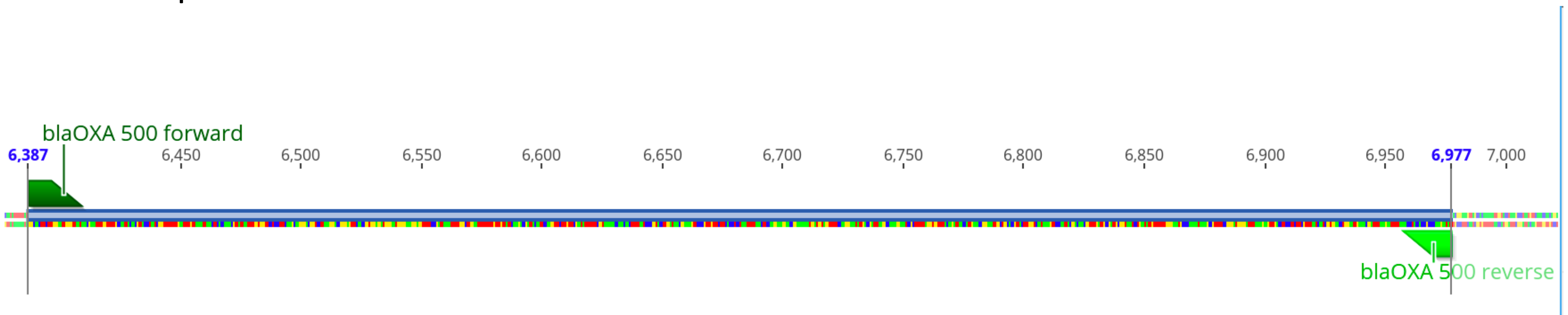

Fig. S3B

<u>Genomic location on Acinetobacter pittii strain IIF1SW-P1</u>

Scaffold 44

GenBank: MIZX01000045.1

Forward: 4018-4037

Reverse: 4407-4388

PCR product expected size: 390 bp

Sequence:

ATGCAGCGCTTCTTTGCAGGTCAGTATTTTGATTATCGTCAAATTTCTCAGTTGATTTTCAATATGTTTT
CATTCGACCAAGTGCAACTGACTTTAGATAGAACCAATTGGAAATGGGGAAAACGAAATATTAATATC
CTGATGCTCGCAATCGTTTATCGTGGAATAGCGATACCTATCCTTTGGACATTGCTTAATAAACGTGGA
AATTCAGATACGAAAGAGCGTATTGCTTTGATTCAACGCTTTATAGCCATTTTTGGTAAAGACCGTATT
GTGAATGTGTTCGCAGACAGAGAGTTTATCGGTGAGCAGTGGTTTACATGGTTAATTGAACAAGACAT
CAACTTCTGCATTCGTGTTAAAAAAACTTCATTGTCACCAATCATT

Primer Map:

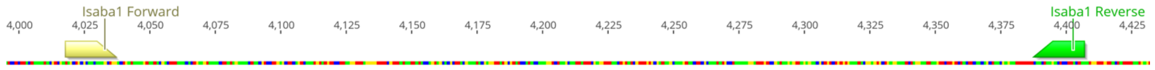

Scaffold 70

GenBank: MIZX01000070.1

Forward: 932-913

Reverse: 543-562

PCR product expected size: 390 bp

Sequence:

AATGATTGGTGACAATAAAGTTTTTTTAACACGAATGCAGAAGTTGATGTCTTGTTCAATTAACCATGT
AAACCACTGCTCACCGATAAACTCTCTGTCTGCGAACACATTCACAATACGGTCTTTACCAAAAATGG
AGATAAAGCGTTGAATCAAAGCAATACGCTCTTTTGTATCTGAATTTCCACGTTTATTAAGTAATGTCC
AAACGATAGGTATCGCTATTCCACGATAAACGATCGCGAGCATCAGGATATTAATATTTCGTTTTCCCC
ATTTCCAATTGGTTCTATCTAAAGTCAGTTGCACTTTGTCGAATGAAAATATATTGAAAATCAACTGAG
AAATTTGACGATAATCGAAATACTGACCTGCAAAGAAGCGCTGCAT

Primer Map:

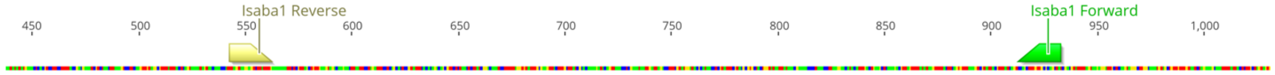

Supplement: Supplementary file 1 [file life-11-00960-s001.zip › life-1372299-supplementary.pdf]
